# Declining trends in Hepatitis A seroprevalence over the past two decades, 1998–2017, in Pune, Western India

**DOI:** 10.1017/S0950268820000953

**Published:** 2020-05-08

**Authors:** Avinash R. Deoshatwar, Yogesh K Gurav, Kavita S Lole

**Affiliations:** 1 Epidemiology Group, ICMR- National Institute of Virology (NIV), 130/1 Sus Road, Pashan Pune 411021, India; 2 Hepatitis Group, ICMR-NIV, 130/1 Sus Road, Pashan, Pune 411021, India

**Keywords:** Declining seroprevalence, hepatitis A, longitudinal data, Western India

## Abstract

Reduction in seroprevalence of Hepatitis A virus (HAV) is known to be associated with improvements in socioeconomic conditions of the community. National Institute of Virology, Pune has been studying seroprevalence of hepatitis viruses in Pune region over the past four decades. In total, 1438 samples were collected from urban general (UGEN), urban lower socioeconomic stratum (ULSES) and rural (RURAL) populations of the Pune district. Based on estimates in previous studies, subjects were enrolled from age groups ‘6–10’, ‘15–25’ and ‘40 + ’ years. HAV seroprevalence in younger population showed a significant decline. A significant decline in HAV seroprevalence in ‘15–25’ years age group in UGEN (from 85.9% to 73.9%; OR = 0.46, 95% CI: 0.25–0.86) and RURAL (from 98.6% to 91.4%; OR = 0.15, 95% CI: 0.05–0.45) populations suggested that the trend probably started more than a decade ago. Seroprevalence of HAV among ULSES ‘6–10’ children was found to be significantly higher (70.4%) than that among the RURAL children (44.2%; OR = 3.0, 95%CI: 1.7–5.2) and UGEN children (40.4%; OR = 3.5, 95%CI: 1.8–6.7). In view of increasing rates of urbanisation in India, ULSES population needs special consideration while designing future studies and viral hepatitis vaccination/elimination strategies. Our findings call for robust population-based studies that consider heterogeneity within populations and dynamics of socio-economic parameters in various regions of a country.

Epidemiology of hepatitis A virus (HAV) varies by socio-economic status of the population as well as with water quality, hygiene and sanitation [1]. Over the last few decades, globally, the seroprevalence of HAV has been in decline in developed as well as in developing countries [2]. India has been experiencing significant socio-economic growth over the last few decades and there are reports indicating a decline in HAV seroprevalence in India during the same period [3, 4]. The decline in seroprevalence is evident initially in the younger age groups, because HAV infections lead to lifelong immunity and, thus, older age groups will carry high seroprevalence due to past exposures [5]. The proportion of asymptomatic/sub-clinical HAV infections is higher among <5 children as compared to older age groups [5]. The decline in seroprevalence in children below 5 years of age paradoxically results in higher burden of clinical disease as hepatitis A disease severity increases with age [1]. It has been seen that once started, the decline in HAV seroprevalence can be rapid, as evidenced by studies from developed as well as developing countries [2]. Considering the rapidity of these changes, for a large country, with complex health and economic diversities like India, robust prevention strategies need evidence to be generated in a longitudinal form.

As per the United Nations Development Program (UNDP) and Maharashtra state government joint report 2012, socioeconomically, Maharashtra is among the top five states in India [6]. The Indian Council of Medical Research (ICMR)-National Institute of Virology (NIV), Pune, India has been generating viral hepatitis seroprevalence data from urban as well as rural areas of this Western district since 1978. HAV seroprevalence from this region was reported by ICMR-NIV in 1982, 1992, 1998 and 2002–05 [3, 7, 8]. HAV seroprevalence assessments were also conducted in 2009 [ICMR-NIV unpublished data]. Socioeconomic parameters of this population have changed over the past two decades. Currently, the private paediatricians are recommending HAV vaccine but there is no evidence available on the extent of vaccine uptake in this region. Elimination of viral hepatitis by 2030 requires recent and robust evidence. We chose this region for study as previous surveys by ICMR-NIV in the same area provide valuable baseline information. In this article, we present findings from the same region in the context of longitudinal data generated over past two decades; with the 1998 study data by Arankalle *et al*., as the reference for comparison [8].

Population divisions were used as they were during the previous studies. Rural overall population (RURAL), urban lower socio-economic strata, (ULSES) and urban general sections (UGEN) of the population were considered. No distinction between higher or lower socioeconomic groups in rural areas was made; in this study as well as in the previous studies [3, 7, 8]. The decline in seroprevalence in ‘6–10’ age group would be indicative of that in the younger age group. Hence, the age group ‘1–5’ years was not considered for feasibility and ethical reasons. Villages from two different blocks in the Pune district, Shirur (North-eastern block in the district) and Bhor (Southern block) were randomly chosen to represent the rural area. Pune city has a population of around 3.1 million (Census 2011, India). ‘Janata Vasahat’ is an urban slum with a population close to 30 000. The slum receives a piped water supply from the city corporation. ‘Janata Vasahat’ slum was chosen to represent urban lower socioeconomic strata (ULSES). While for the urban general population, schools, colleges, institutions and offices in the Pune city were considered for the recruitment of the participants. Written informed consents were obtained from adult participants. For minors, the consent was obtained from parents. Proforma that recorded demographics, housing conditions, history of jaundice, water sources and other risk factors for viral hepatitis was administered. Serum samples, 2–5 ml, were collected with aseptic precautions by trained technicians from consenting individuals/wards of consenting parents. The sample collections were carried out at respective schools/colleges/offices of the participants. In the current study, serum samples were screened for the presence of IgG antibodies against hepatitis A virus (HEPAVASE A-96, anti-HAV IgG ELISA kit, General Biologicals Corporation, Taiwan).

Age group-wise HAV seroprevalence values from 1998 study were taken as the reference to calculate sample sizes for the age groups, 6–10, 16–25 and 40 + years. The sample size was calculated to detect an assumed 10% reduction in seroprevalence, over two decades, by chi-squared analysis. Data were collected on proforma and entered into MS Excel 2007. Simple proportional analyses were performed with the help of Epi Info-7 software (CDC, Atlanta) to compare seroprevalence across age groups and socio-economic groups.

Simple proportional analyses were performed to understand the change in HAV seroprevalence over two decades; age-group wise findings were compared to those from 1998 serosurvey (abstracted from Arankalle *et al*., [8]).

All respondents did not answer all the questions in the proforma. The data were analyzed assuming that the missing data did not introduce any selection bias.

A total of 767 samples were collected from rural areas of the Pune district, 285 in the ‘6–10’ years age group, 313 in the ‘15–25’ years age group and 169 in the ‘40 + ’ years age group. From the urban region, a total of 671 samples were collected; 170 from ‘6–10’ years, 291 from ‘15–25’ years and 180 from ‘40 + ’ years age groups.

A significant reduction was noted in ULSES as well as RURAL populations in ‘6–10’ age group; however, UGEN population showed a statistically insignificant increase in the same age group. Also, a significant reduction was seen in the overall ‘15–25’ age group (Table 1, Part A). The seroprevalence in ‘40 + ’ age group did not show any decline (98.8% in RURAL, 96.52% in UGEN and 100% in ULSES populations in 2017).

**Table 1.** Comparative HAV seroprevalence in rural and urban areas and sources of drinking water in rural populations, Pune district, Western India, 1998 and 2017

Part A: Comparative HAV seroprevalence between 1998 and 2017									
Area of residence	Positive/number tested (%)								
	Urban						Rural		
	Urban General			Urban Low SES^#^					
**Age group**	**1998**^a^	**2017**	**OR**^b^	**1998**	**2017**	**OR**	**1998**	**2017**	**OR**
**6–10**	67/217 (30.9%)	40/99 (40.4%)	1.51 (0.93–2.49)	483/514 (94%)	50/71 (70.4%)	**0.15 (0.08–0.29)**	528/571 (92%)	126/285 (44.2%)	**0.06 (0.04–0.10)**
**16–25**	171/199 (85.9%)	68/92 (73.9%)	**0.46 (0.25–0.86)**	198/198 (100%)	167/199 (83.9%)	**0.00 (Undefined)**	275/279 (98.6%)	286/313 (91.4%)	**0.15 (0.05–0.45)**
**40+**	**-**	63/63 (100%)	**-**	**-**	114/117 (97.4%)	-	**-**	167/169 (98.2%)	-
**Total (For 6–10 And 16–25)**	238/416 (57.2%)	108/191 (56.5%)	0.97^c^ (0.69–1.37)	681/712 (95.6%)	217/270 (80.4%)	**0.19 (0.17–0.30)**	803/850 (94.5%)	412/598 (68.9%)	**0.13 (0.09–0.18)**

Part B: HAV seroprevalence among rural population by drinking-water source Pune, 2016–17.			
Water Source	Users (% among respondents)	Anti- HAV Positive(n)	(%)
Bore well	155 (20.4)	127	(81.9)
Common community source	16 (2.1)	13	(81.3)
Tap water	235 (30.9)	182	(77.4)
Water filter at home	125 (16.4)	**66**	**(52.8)**
Bottled water	1 (0.13)	1	(100.0)
Protected well	85 (11.2)	81	(95.3)
Unprotected well	20 (2.6)	17	(85.0)
Mixed	4 (0.52)	4	(100.0)
Source not reported	119 (15.7)	87	(73.1)
Total	760	578	(76.1)

^#^Low Socioeconomic status.

The confidence intervals for ORs indicate the statistical significance of the bold values.

a 1998 Reference values abstracted from article by Arankalle *et al*., [8].

b Odds ratios (95% Confidence Interval).

c OR shows insignificant decline because contrary to assumptions, the HAV seroprevalence has increased (though statistically insignificant) in the ‘6–10 years’ age group in the urban general population over the period of 20 years from 1998 to 2017; which can be attributed to vaccination provided by private healthcare providers.

Seroprevalence in ULSES ‘6–10’ children was significantly higher than that in rural children; on the other hand, in ‘15–25’ years age group, it was significantly higher in RURAL than USLES (OR = 2.04, 95% CI: 1.18–3.45), indicating a sharper decline in HAV seroprevalence in the recent past in rural areas. In the present serosurvey, age-dependent increase was seen for HAV in rural as well as urban ULSES and UGEN population groups.

Drinking water and HAV seroprevalence: In rural areas, HAV seroprevalence was significantly lower among subjects who reported the use of commercially available water filter at home than among those who did not (OR = 0.25, 95%CI: 0.16–0.37). Use of tap water, bore-well water or protected/unprotected well as the drinking-water source did not have a significant effect on HAV seroprevalence. (Table 1, Part B)

Of the 837 subjects who responded to the ‘history of jaundice’ question, 72 (8.6%) subjects responded positively. (Table 1, Part B) Among these, 12 (16.7%) were in the ‘6–10’ years age group, 45 (62.5%) in ‘15–25’ years and 15 (20.8%) in ‘40 + ’ age group. Of the total 72 subjects, 68 were positive for anti-HAV antibodies. Among the 72 subjects with a history of jaundice, none were positive for anti-HCV or HBsAg. Odds of the people with a history of jaundice to be positive for HAV infection were significantly high. There were four subjects who reported a history of jaundice but were negative for HAV and HEV; three of the subjects were from ‘6 to 10’ years age group while one was a young adult between ‘15 and 25’.

Our findings indicate that it is time to generate robust and timely evidence to design well-directed policies for attaining viral hepatitis elimination goals by 2030. Developing countries, e.g. Brazil, Greece and China that experienced a transition in HAV have introduced vaccine against the virus in their national immunisation programs [9–11]. National Technical Advisory Group on Immunisation [12] (NTAGI) had emphasised the need for robust epidemiological data for making policy decisions [12, 13]. In hyperendemic regions, the reduction in HAV seroprevalence in any population over time is first reflected in the youngest population. This is because >95% members of such populations are seropositive by the age of 5 years.

We compared our results to those of the 1998 serosurvey in the same region. In the urban 15–25 years age group, there was a significant reduction in HAV exposure in both ULSES and UGEN populations in 2017 as compared to 1998, indicating that this trend is not very recent and probably started more than a decade ago in the urban area (Table 1, Part A). For the urban general population HAV seroprevalence in 2017 in the ‘6–10’ age group was marginally higher than that in 1998, probably because of vaccination by private healthcare providers. HAV vaccination, even in the private sector, is a recent phenomenon; hence HAV seroprevalence is high in children but has reduced significantly in young adults among this section of the urban population (Table 1, Part A). Reasons for these unequal trends among children and young adults need to be explored. Significant reduction in exposures of both 6–10 years and 15–25 years age groups in ULSES section further supported that there is a declining trend more than a decade ago in the urban area. The data (Table 1, Part A) indicate that trend of reduction in HAV seroprevalence started earlier among the urban population than the rural. In rural population, the decrease is marked in ‘6–10’ years age group while there is no change among the young adults.

Not all participants in the study responded to all the questions in the proforma. We believe that it did not introduce any selection bias and analyzed the data accordingly. This, however, is a statistical limitation of these analyses. Data on history of jaundice may be limited by recall bias, though the authors believe that people generally tend to remember if they had jaundice in the past. The study area was chosen because it was the only region in this part of the country from which longitudinal hepatitis seroprevalence data were available. Similar multi-centric studies are required in order to understand seroprevalence levels across the country. Another possible limitation could be that the two surveys were used in two different assays. The sensitivity and specificity of the HAV IgG kits used for the 2017 survey is 99.8% and 99.5%, respectively. Anti-HAV antibody detection in 1998 was done by using an in-house assay developed at the ICMR-National Institute of Virology. This assay was compared with HAV AB EIA and RIA, Abbott Laboratories, USA and was found to have 100% sensitivity and specificity [14]. Although the two studies used different assays, the two assays were comparable.

Among subjects with a history of jaundice, evidence of past HAV infection was noted in 68 of 72 subjects, of these, 20 were also positive for HEV. All HEV positive subjects with a history of jaundice were also positive for HAV. The odds of subjects with jaundice to be positive for HAV were significantly higher than those without it (OR = 4.65, 95%CI: 1.67–12.93); in other words, in the absence of other causes of hepatitis, HAV appears to be the major cause of jaundice in this area. With a marked reduction in childhood seroprevalence, the HAV disease burden is likely to increase.

When overall HAV seroprevalence in the ‘6–10’ and ‘15–25’ years age groups was compared from 1978 to 2017 by taking previously reported values from NIV [7, 8], a marked decline was observed over the last two decades while no decline was seen over two decades before 1998. However, when compared with 2009 seroprevalence [NIV unpublished data] the decline in 2017 was seen to be rapid during the last decade (from ~74% to ~44%). The decline was prominent among children while it was not noticed among young adults. The WHO advocates the categorisation of populations according to HAV immunity rates in the community [15].This population seems to have moved rapidly from ‘high child immunity rates’ (>90%) to ‘low-medium immunity rates’ (40%–59%). It has been reported from South-east Asian countries that the transitions in HAV seroprevalence can be rapid [15]. Rapid changes pose significant challenges to policy formulation.

The ‘adult susceptibility rate’ (proportion of non-immune adults between 35 and 44 years) has remained very low (close to 0%) over the past two decades.

This effort highlights a significant reduction in HAV seroprevalence in this region over the last two decades. The decline has been rapid which is in agreement with trends observed in other developing countries. It appears that urban lower socioeconomic sections need special consideration while designing prevention strategies, especially in the light of the increasing speed of urbanisation. The HAV elimination strategies need to consider the heterogeneity of populations and the dynamics of socio-economic situations. Similar efforts in other parts of the country, through multi-centric studies, will help the efforts to achieve the goal of viral hepatitis elimination in India by 2030.
